# Calcium Silicate-Based Cements for Vital Pulp Therapy: Integrated Assessment of Radiopacity, Elemental Composition, and 24-h Pulp Cell Responses

**DOI:** 10.3390/biomimetics11040280

**Published:** 2026-04-17

**Authors:** Belen Şirinoğlu Çapan, Vasfiye Işık, Tugba Elgün, Zeynep Hale Keleş, Soner Şişmanoğlu

**Affiliations:** 1 Department of Pediatric Dentistry, Faculty of Dentistry, Istanbul University-Cerrahpasa, Istanbul 34098, Türkiye; belen.capan@iuc.edu.tr; 2 Department of Endodontics, Faculty of Dentistry, Istanbul University-Cerrahpasa, Istanbul 34098, Türkiye; vasfiye.isik@iuc.edu.tr; 3 Department of Medical Biology, Faculty of Medicine, Biruni University, Istanbul 34015, Türkiye; 4 Department of Restorative Dentistry, Faculty of Dentistry, Istanbul Atlas University, Istanbul 34408, Türkiye; 5 Department of Restorative Dentistry, Faculty of Dentistry, Istanbul University-Cerrahpasa, Istanbul 34098, Türkiye

**Keywords:** radiopacity, EDX, cytotoxicity, cytokine expression, 24-h assessment, calcium silicate cements, vital pulp therapy

## Abstract

This study investigated the radiopacity, elemental composition, cytotoxicity, and cytokine responses of contemporary calcium silicate-based cements containing different radiopacifiers. Four cement materials (NeoMTA2, NeoPUTTY, TheraCal PT, and One-Fil PT) were evaluated. Radiopacity was measured using digital radiography with a 10-step aluminum wedge and expressed in mm Al in accordance with ISO 6876; among three calibration models compared, the quadratic provided the best fit. Elemental composition was analyzed by SEM/EDX. Cytotoxicity was assessed on human dental pulp cells using the MTT assay, and IL-6 and IL-10 levels were quantified by ELISA. One-Fil PT (6.61 mm Al) and NeoPUTTY (6.09 mm Al) showed the highest radiopacity, whereas TheraCal PT (1.61 mm Al) did not meet ISO standards. SEM/EDX revealed tantalum in NeoMTA2 and NeoPUTTY, and zirconium in One-Fil PT and TheraCal PT. NeoPUTTY and NeoMTA2 demonstrated superior cell viability, while One-Fil PT showed the lowest. TheraCal PT and One-Fil PT increased IL-6 expression, whereas NeoPUTTY and NeoMTA2 promoted higher IL-10 levels. Within the limitations of this 24-h in vitro assessment, NeoMTA2 and NeoPUTTY exhibited more favorable short-term cytocompatibility and inflammatory profiles together with adequate radiopacity. These findings require confirmation through long-term in vivo and clinical studies.

## 1. Introduction

Calcium silicate-based cements are now routinely used in vital pulp therapy (VPT) and root canal management applications. The widespread adoption of these materials in clinical practice stems from their bioactivity, superior sealing ability, and reliable clinical performance. First exemplified by mineral trioxide aggregate (MTA), these cements exhibit well-documented interactions with hard tissues, promoting odontoblastic differentiation, hydroxyapatite formation, and reparative dentin deposition [1,2].

Studies conducted over the past twenty years have focused on overcoming the various limitations of traditional MTA formulations. These include long setting time, difficult handling characteristics, discoloration potential, and impurity issues related to the presence of bismuth oxide and heavy metals [3]. These limitations have led to the development of new-generation calcium silicate-based cements, including ready-to-use putty-type calcium silicate-based cements.

VPT is a well-established approach in modern dentistry that aims to preserve pulp vitality following mechanical or carious exposures through the formation of a mineralized barrier in the exposure area. Calcium silicate-based cements are widely used in VPT procedures because they stimulate hard tissue formation, create an alkaline environment that promotes healing, and exhibit effective sealing properties [4,5,6]. The success of VPT largely depends on the biocompatibility and bioactivity properties of the materials used. This has created a demand for advanced formulations that offer low cytotoxicity alongside adequate physicochemical performance.

Changes in the selection of radiopacifying agents have been particularly effective in optimizing material performance. Bismuth oxide, traditionally used to provide adequate radiopacity, has been associated with tooth discoloration, altered hydration kinetics, and potential cytotoxicity [7,8]. These concerns have led researchers to explore alternative radiopacifiers. Zirconium oxide, tantalum oxide, and calcium tungstate are prominent alternatives in this context. Comparative analyses have shown that zirconium oxide can provide radiopacity levels similar to bismuth oxide, while accomplishing this without affecting cement hydration or causing discoloration [9]. Calcium tungstate, on the other hand, meets international radiopacity standards while improving optical stability and handling properties [10,11]. Radiopacifier selection therefore involves a trade-off between physicochemical and biological properties that must be carefully considered for each formulation.

The biological behavior of calcium silicate-based cements may be influenced by differences in material composition in addition to radiopacity-related characteristics. For example, calcium ion release activates signaling pathways that contribute to mineralized tissue formation and pulp healing processes [12]. However, differences in additives such as plasticizers, resin components, and radiopacifiers can alter pH, solubility, and bioactivity, sometimes leading to inflammatory or cytotoxic responses in vitro [13,14]. Cytocompatibility testing is accordingly an essential step in the preclinical evaluation of any new formulation. Furthermore, research on the genotoxic and oxidative effects of new formulations further reinforces the case for thorough biocompatibility screening [15,16]. Studies on human dental pulp stem cells have shown that premixed calcium silicate-based cements generally support cell viability, migration, and odontogenic differentiation, although the extent of these responses varies depending on material composition and the presence of resin components [6,17,18]. However, concerns have been raised regarding the biocompatibility of resin-modified formulations, as these materials have been associated with increased cytotoxicity, incomplete hydration, and a shift toward pro-inflammatory responses compared to conventional calcium silicate-based cements [17,19].

Advances in pre-mixed calcium silicate-based cements, including putty materials, aim to provide more consistent handling properties and controlled hydration reactions. The ready-to-use nature of these materials eliminates operator-dependent variability and provides clinical advantages in pediatric and adult endodontics by limiting moisture exposure during manipulation [20]. Current studies show that these materials generally exhibit excellent sealing ability and favorable cell viability; however, performance differences may be observed depending on factors such as the type and concentration of radiopacifier used, fillers, and thickening agents [20,21]. A combined evaluation of physicochemical and biological properties is therefore necessary to establish the clinical suitability of new putty-type calcium silicate-based cement formulations. Recent clinical evidence has further supported the use of premixed calcium silicate-based cements in vital pulp therapy and related applications. Favorable clinical outcomes, including high success rates and predictable hard tissue barrier formation, have been reported with contemporary premixed formulations in both pulpotomy and direct pulp capping procedures [22,23]. Prospective evaluations have also demonstrated favorable healing rates and tooth survival with premixed calcium silicate-based cement formulations in broader clinical settings [24]. These findings reinforce the clinical relevance of evaluating the physicochemical and biological properties of current premixed calcium silicate-based cements intended for pulp therapy applications.

Recent classification frameworks have categorized calcium silicate-based cements into distinct generations based on their compositional evolution and setting mechanisms [19,25]. Among these, resin-modified calcium silicate cements (RMCSCs), such as TheraCal LC and TheraCal PT (Bisco, Schaumburg, IL, USA), constitute a separate category (Generation VI) characterized by the incorporation of a polymerizable resin matrix. Unlike conventional calcium silicate-based cements that set exclusively through hydraulic reactions, RMCSCs rely on light-cure or dual-cure polymerization, with limited subsequent hydration dependent on environmental moisture uptake. This fundamental difference in setting chemistry has been associated with altered biological responses, including reduced calcium ion release and lower biocompatibility compared to earlier-generation calcium silicate-based cements. The present study therefore includes both calcium silicate-based cements and an RMCSC to capture the influence of these compositional and mechanistic differences on radiopacity, elemental composition, and biological behavior.

Despite all advances in the field of pre-mixed calcium silicate-based cements and radiopacifier selection, comprehensive data evaluating radiopacity, elemental composition, and biological behavior together for calcium silicate-based cements in putty form recently introduced to the market are limited. Although individual properties such as cytocompatibility or radiopacity have been studied separately, the interrelationship between chemical composition, inflammatory response, and radiographic performance has not been systematically investigated. This knowledge gap makes evidence-based cement selection difficult in VPT applications. Therefore, the present study aims to comparatively evaluate selected current putty-form calcium silicate-based cements containing different radiopacifiers and formulations in terms of radiopacity, elemental composition, cytotoxicity, and anti-inflammatory effects. The null hypothesis was that there would be no significant difference between the tested putty-form calcium silicate-based cements in terms of radiopacity (according to ISO 6876 [26]requirements) and biological responses (including cytotoxicity and cytokine expression) evaluated on human dental pulp cells.

## 2. Materials and Methods

### 2.1. Study Design

Four different cements, including three calcium silicate-based cements, namely NeoMTA2 (Avalon Biomed, Houston, TX, USA), NeoPUTTY (Avalon Biomed, Houston, TX, USA), and One-Fil PT (Mediclus, Cheongju, Republic of Korea), and one RMCSC, namely TheraCal PT, were included in the study. The compositions and manufacturer information of the materials are presented in Table 1.

The sample size for radiopacity assessment was determined using G*Power software (version 3.1.9.7, Heinrich-Heine-Universität Düsseldorf, Düsseldorf, Germany). The power analysis was performed based on previous radiopacity studies of calcium silicate-based cements, with α = 0.05, power = 0.80, and effect size f = 0.50 for one-way ANOVA. The analysis indicated that a minimum of 12 samples were required for each material. To ensure sufficient statistical power, fifteen samples (*n* *=* 15) were prepared from each material for the radiopacity test.

**Figure 1:**
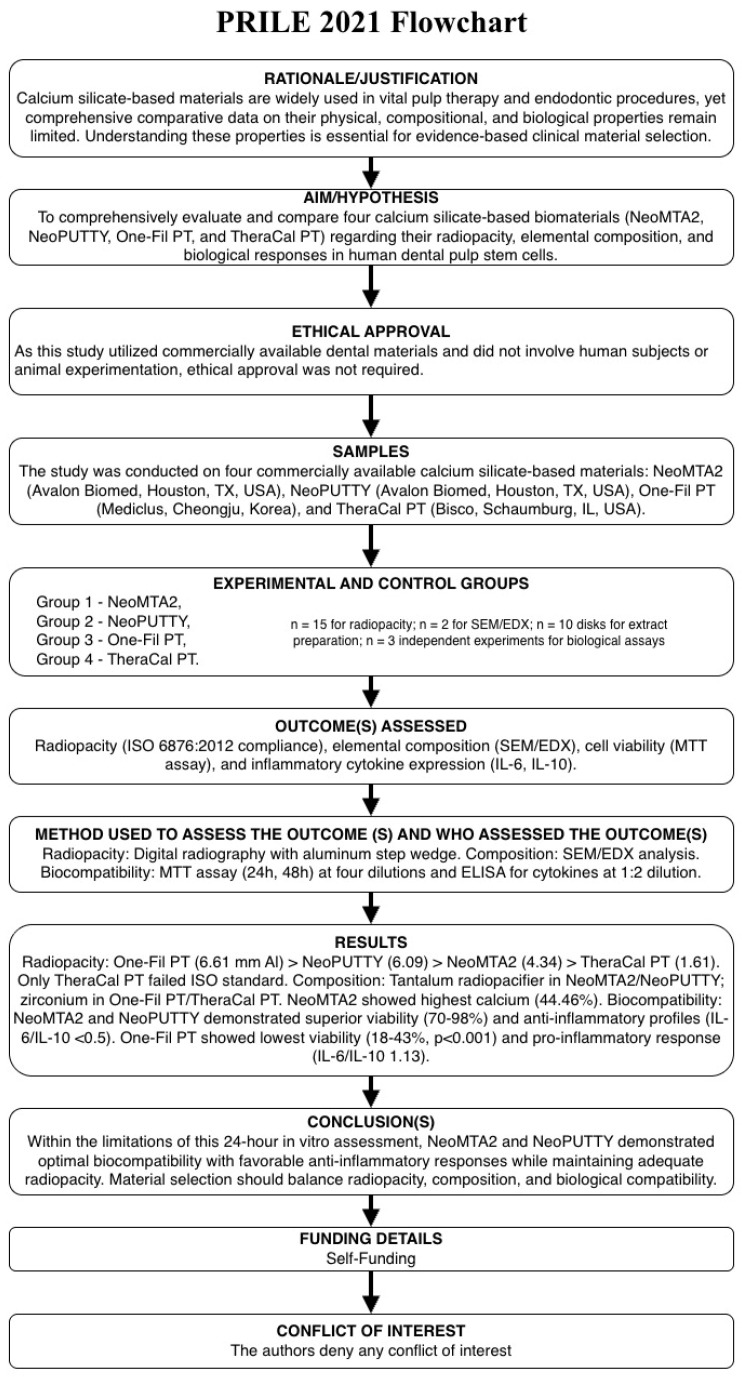
PRILE 2021 flowchart of the experimental design.

The study included radiopacity evaluation according to ISO 6876 (ISO 6876:2012, subsequently revised as ISO 6876:2025; the minimum radiopacity requirement of ≥3 mm Al remains unchanged in the current edition), scanning electron microscopy with energy-dispersive X-ray spectroscopy (SEM/EDX) analysis, cytotoxicity assessment using the MTT assay at four different dilutions (1:1, 1:2, 1:4, 1:8), and inflammatory cytokine expression analysis (IL-6 and IL-10) using enzyme-linked immunosorbent assay (ELISA). Material extracts were prepared according to ISO 10993-5 [27] guidelines. The study design is presented in Figure 1 as a Preferred Reporting Items for Laboratory Endodontology (PRILE) 2021 flowchart. Each material was prepared according to the manufacturer’s instructions. NeoPUTTY, One-Fil PT, and TheraCal PT were used as supplied (premixed, ready-to-use formulations). NeoMTA2 powder was mixed with the accompanying liquid in a 1:1 powder-to-liquid ratio by weight.

### 2.2. Radiopacity Analysis

**Figure 2:**
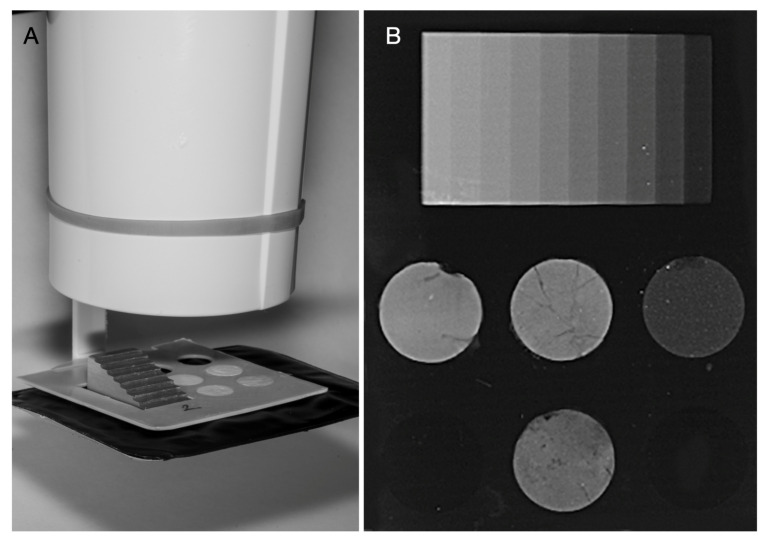
Standardized radiopacity evaluation using custom-fabricated positioning system. (**A**) Three-dimensionally printed apparatus designed to maintain consistent source-to-film distance and specimen positioning, mounted to the X-ray cone. The system incorporates a 3D-printed specimen holder containing test materials and aluminum step wedge, positioned above an occlusal radiographic film. (**B**) Representative radiographic image demonstrating the aluminum step wedge reference (top) and four tested material groups, obtained using the standardized geometric configuration to ensure reproducible radiopacity measurements according to ISO 6876.

For radiopacity assessment, a positioning apparatus was specifically designed using TinkerCAD (Autodesk Inc., San Rafael, CA, USA) and 3D-printed using a Bambu Lab A1 FDM printer (Bambu Lab, Shenzhen, China) with polylactic acid (PLA) filament. The apparatus consisted of two components: (1) a specimen holder containing standardized cylindrical cavities (10 mm diameter, 1 mm depth) for material samples and a special slot for the aluminum step wedge, and (2) a mounting adapter that allows the holder to be attached to the X-ray cone in a repeatable manner, creating a standardized geometry between the X-ray source, specimens, and detector (Figure 2A).

Cylindrical specimens (10 mm diameter, 1 mm thickness, *n =* 15 for each material) were prepared and placed in the specimen holder together with a 10-step aluminum step wedge (99% purity, 1-mm increments, 1–10 mm thickness range, each step measuring 2.5 mm in width and 15 mm in length) compliant with ISO 6876 standards (Figure 2B). The positioning apparatus maintained a fixed focus-to-detector distance of 30 cm and ensured perpendicular beam alignment.

Digital radiographs were obtained using an intraoral X-ray unit (RXDC Extend; MyRay, Bicocca, Italy) under standardized exposure conditions (70 kVp, 8 mA, 0.32 s). The images were captured using an image plate scanner (VistaScan; Dürr Dental, Bietigheim-Bissingen, Germany) and saved in TIFF format to preserve image quality for quantitative analysis.

Radiographic density was quantified using ImageJ(Mac OS X; version 1.54) software (Fiji distribution, National Institutes of Health, Bethesda, MD, USA). For each specimen and aluminum step, a standardized circular region of interest (ROI, approximately 2 mm in diameter) was defined at the center of each specimen and aluminum step, ensuring that the ROI remained within the step boundaries to minimize potential edge effects and X-ray scattering artifacts, and the average gray value was recorded. The average gray values of the aluminum steps were plotted against their corresponding thicknesses. Three regression models (linear, quadratic, and logarithmic) were compared to determine the most appropriate calibration curve. The quadratic model yielded the highest coefficient of determination (R^2^ = 0.999) compared with the linear (R^2^ = 0.976) and logarithmic (R^2^ = 0.970) models and was therefore selected for converting gray values to aluminum thickness equivalents.

The radiopacity of each material was determined by converting the specimen gray values to aluminum thickness equivalents (mm Al) using the quadratic regression equation obtained from the calibration curve. Results are expressed as mean ± standard deviation. According to ISO 6876, materials used near dental tissues must exhibit a minimum radiopacity of 3 mm Al to provide sufficient radiographic distinction from surrounding anatomical structures. The radiopacity assessment methodology was consistent with protocols previously applied to premixed calcium silicate-based cements [28,29].

### 2.3. Elemental Analysis

For surface characterization, disk-shaped specimens of the same dimensions (10 mm diameter, 1 mm thickness, *n =* 2 for each material) were prepared. After complete setting, the specimens were sputter-coated with gold to provide electrical conductivity and examined using scanning electron microscopy (SEM; Thermo Scientific Apreo S, Waltham, MA, USA). Representative micrographs at 5000× magnification were obtained to document surface morphology, particle distribution, and microstructural characteristics.

Elemental composition was determined using energy-dispersive X-ray spectroscopy (EDX; Thermoscientific Apreo S, Waltham, MA, USA) integrated with the SEM system. Quantitative elemental analysis was performed on the total area of each specimen using EDAX Team software (EDAX Inc., Mahwah, NJ, USA). The weight percentages (wt%) of major elements, including calcium (Ca), silicon (Si), oxygen (O), and radiopacifying agents, were determined, and EDX mapping was performed to evaluate the elemental distribution on the material surface. In the present study, EDX analysis was used as a targeted compositional assessment to identify the principal inorganic and radiopacifying elements relevant to the study aim, rather than to provide a full surface chemical characterization. The analysis was repeated twice for each sample.

### 2.4. Preparation of Material Extracts and Cells for Cell Culture

All materials were prepared under aseptic conditions and placed into sterile cylindrical molds. Following preparation, the samples were sterilized with ultraviolet (UV) irradiation for 1 h and then incubated at 37 °C for 24 h to ensure complete setting.

Stock extracts were prepared in accordance with ISO 10993-5 guidelines. For preparation of the stock extract, 10 disk specimens from each material were immersed in 5 mL of Dulbecco’s modified Eagle’s medium (DMEM; Sigma-Aldrich, St. Louis, MO, USA) and incubated at 37 °C for 24 h in a humidified atmosphere containing 5% CO_2_, maintaining a surface area-to-medium volume ratio of approximately 1.1 cm^2^/mL in accordance with the relevant ISO standard. Following incubation, the extraction media were filtered through sterile 0.22 μm syringe filters. Serial dilutions of 1:1 (100%), 1:2 (50%), 1:4 (25%), and 1:8 (12.5%) were prepared from the stock extracts using fresh DMEM for subsequent biological assays.

Human dental pulp stem cells (hDPSCs; CELPROGEN, 36086-01, Torrance, CA, USA) were cultured in DMEM supplemented with 10% fetal bovine serum (FBS), 100 U/mL penicillin-streptomycin, and L-glutamine. Cells were maintained at 37 °C in a humidified atmosphere containing 5% CO_2_.

### 2.5. Determination of Cell Viability and Inflammation Markers

The cytotoxic effects of the four test materials on cell viability were evaluated using the 3-(4,5-dimethylthiazol-2-yl)-2,5-diphenyltetrazolium bromide (MTT) assay kit (Abcam, Cambridge, UK). The MTT assay was selected in the present study due to its widespread use, reproducibility, and reliability in evaluating cell viability and cytotoxicity in dental material research, and despite its known limitations, such as dependence on cellular metabolic activity and potential chemical interference, it remains one of the most commonly used methods for comparative cytotoxicity assessment, particularly in studies involving pulp-related cells [30]. Cells from the third passage were seeded at a density of 2 × 10^4^ cells/well into wells containing complete DMEM (Dulbecco’s Modified Eagle Medium supplemented with 10% fetal bovine serum and 100 U/mL penicillin–streptomycin) in 96-well plates. To allow cell adhesion, after 24 h of incubation at 37 °C in a humidified atmosphere with 5% CO_2_, the culture medium was replaced with material extracts at 1:1, 1:2, 1:4, and 1:8 dilutions or fresh DMEM as a negative control. Different well groups were evaluated after a 24-h exposure period. Following exposure, the culture medium was carefully removed, and MTT reagent was added (0.5 mg/mL) to each well, and the plates were incubated for 4 h to allow formazan crystal formation. After incubation, the supernatant was discarded, and the resulting formazan crystals were solubilized using dimethyl sulfoxide (DMSO). Absorbance was measured at 590 nm at 24 h by using a microplate reader. Cell viability was expressed as a percentage relative to the untreated control group. Each experiment was repeated three times.

IL-6 and IL-10 levels were quantified using commercially available human ELISA kits (BT LAB, Shanghai, China). Cells from the third passage were seeded at a density of 5 × 10^4^ cells/well into wells containing complete DMEM in 24-well plates. After 24 h of incubation to allow cell adhesion, the culture medium was replaced with material extracts diluted 1:2, representing a moderate exposure condition. Different well groups were evaluated after a 24-h exposure period. Following exposure, culture supernatants were collected, and IL-6 and IL-10 concentrations were measured according to ELISA kit protocols. Each experiment was repeated three times.

### 2.6. Statistical Analysis

All statistical analyses were performed using SPSS software (version 31.0; IBM Corp., Armonk, NY, USA). Data are presented as mean ± standard deviation. For radiopacity measurements (*n =* 15 per group), normality was verified using the Shapiro–Wilk test and homogeneity using Levene’s test. One-way analysis of variance (ANOVA) was applied to compare groups, followed by Tukey’s post-hoc test for pairwise comparisons. For cell viability (MTT assay) and cytokine expression (ELISA) experiments (*n =* 3 per group), the Kruskal–Wallis test was used to compare materials, followed by Mann–Whitney U tests with Bonferroni correction for pairwise comparisons. For energy-dispersive X-ray spectroscopy (EDX) analysis, elemental composition data are presented as descriptive statistics (mean ± standard deviation). Statistical significance was set at *p <* 0.05 for all analyses.

## 3. Results

### 3.1. Radiopacity Analysis

**Figure 3:**
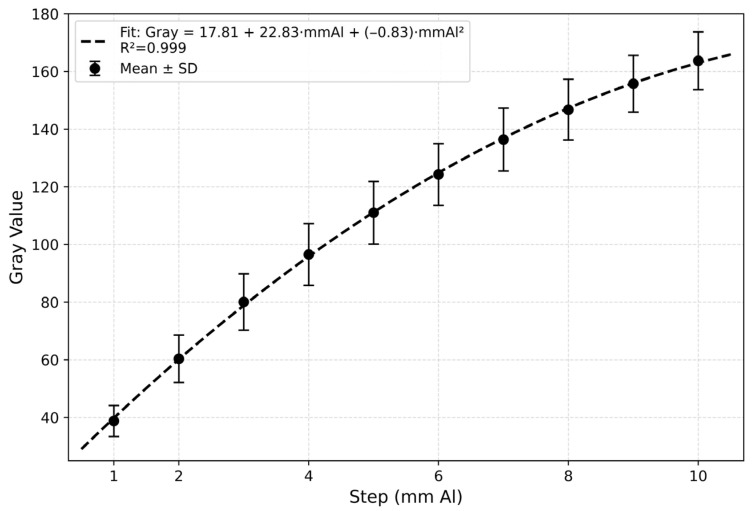
Calibration curve obtained from the aluminum step-wedge (1–10 mm) across radiographs. Data are presented as mean gray values ± standard deviation for each step. The dashed line represents the quadratic regression fit (Gray = 17.81 + 22.83·mmAl − 0.83·mmAl^2^, R^2^ = 0.999), which was used to convert sample gray values into aluminum thickness equivalents (mm Al).

The calibration curve obtained from the aluminum step wedge showed a strong relationship between aluminum thickness and mean gray values. Among the regression models compared (linear, quadratic, and logarithmic), the quadratic model provided the best fit (Gray = 17.81 + 22.83·mmAl − 0.83·mmAl^2^, R^2^ = 0.999) and was therefore used to convert sample gray values to aluminum thickness equivalents (mm Al) (Figure 3). This regression equation was used to convert the sample gray values to aluminum equivalents (mm Al).

The average radiopacity values of the tested materials are presented in Table 2. NeoMTA2 (4.34 ± 0.42 mm Al), NeoPUTTY (6.09 ± 0.54 mm Al), and One-Fil PT (6.61 ± 0.32 mm Al) showed the highest radiopacity, while TheraCal PT (1.61 ± 0.21 mm Al) exhibited the lowest value. One-way ANOVA revealed statistically significant differences between groups (F = 490.5, *p <* 0.001). Tukey’s post hoc test showed that all pairwise comparisons were statistically significant (*p <* 0.05), and the ranking was as follows: One-Fil PT > NeoPUTTY > NeoMTA2 > TheraCal PT. When evaluated for compliance with ISO 6876, NeoMTA2, NeoPUTTY, and One-Fil PT met the minimum 3 mm Al requirement, while TheraCal PT did not meet this threshold.

### 3.2. Elemental Analysis

The elemental composition results determined by energy-dispersive X-ray spectroscopy revealed distinct differences among the tested materials (Table 3: weight%, Table 4: atomic%). The four calcium silicate-based cements exhibited different profiles, particularly in terms of radiopacifying agents and silicon content.

In terms of radiopacifying elements, NeoMTA2 and NeoPUTTY were found to contain tantalum as the main radiopacifying agent (average weight percentage: 0.32 ± 0.51 and 5.71 ± 5.72, respectively). One-Fil PT and TheraCal PT, on the other hand, contained zirconium as their radiopacifier at substantially higher concentrations (30.59 ± 1.75% and 26.42 ± 1.76%, respectively). Additionally, barium (2.12 ± 0.74 wt%) was detected in the structure of TheraCal PT as a supplementary radiopacifying element. The low tantalum content in NeoMTA2 (0.32 wt%) was consistent with its minimal atomic proportion (0.04 at%) due to tantalum’s high atomic weight compared to other elements.

Silicon content showed significant variation, reflecting the different formulation strategies of the materials. TheraCal PT had the highest silicon concentration (16.14 ± 4.02 wt%, 14.87 ± 3.77 at%), and this finding was consistent with the material’s resin-modified calcium silicate composition. NeoPUTTY exhibited moderate silicon values (7.20 ± 2.58 wt%, 5.97 ± 2.49 at%), while NeoMTA2 showed lower silicon content (3.04 ± 0.78 wt%, 2.41 ± 0.61 at%). One-Fil PT, on the other hand, revealed a predominantly pure calcium silicate matrix structure with minimal silicon content (0.75 ± 0.27 wt%, 0.77 ± 0.28 at%).

Calcium concentrations showed significant differences between cements. NeoMTA2 had the highest calcium content (44.46 ± 1.76 wt%, 24.77 ± 1.24 at%), followed by NeoPUTTY (36.30 ± 3.15 wt%, 20.72 ± 0.56 at%) and One-Fil PT (31.29 ± 2.48 wt%, 22.44 ± 1.76 at%). TheraCal PT exhibited a significantly lower calcium content (12.51 ± 5.26 wt%, 8.06 ± 3.37 at%). This lower calcium content likely reflects dilution by the resin components in the formulation.

The oxygen content ranged from 37.37 ± 0.55 wt% (One-Fil PT) to 52.18 ± 1.36 wt% (NeoMTA2), while NeoPUTTY and TheraCal PT showed intermediate values of 50.79 ± 4.99 wt% and 42.80 ± 1.02 wt%, respectively. When evaluated on an atomic basis, oxygen was the predominant element in all materials, ranging from 67.15 to 72.78 at%. This reflects the oxide-based nature of calcium silicate chemistry. Oxygen’s relatively narrow atomic percentage range (5.63 at% difference) contrasted with the wider weight percentage distribution (14.81 wt% difference), highlighting the strong influence of heavier elements on the gravimetric composition.

**Figure 4:**
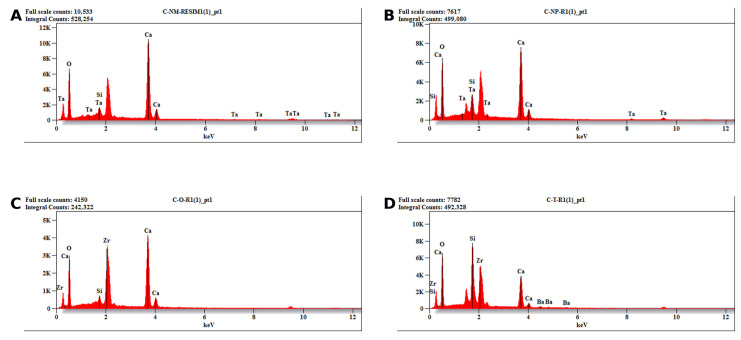
Representative full energy-dispersive X-ray spectroscopy (EDX) spectra of (**A**) NeoMTA2, (**B**) NeoPUTTY, (**C**) One-Fil PT, and (**D**) TheraCal PT are shown. The spectra illustrate the principal inorganic and radiopacifying elements detected in each material. These spectra are presented as representative qualitative profiles, while the quantitative elemental data are reported separately in Table 3 and Table 4.

Standard deviation values showed variability in the detected elemental composition among the tested materials. NeoPUTTY exhibited higher variation in tantalum (SD = 5.72) and oxygen (SD = 4.99) values, while TheraCal PT showed variation in calcium (SD = 5.26) and silicon (SD = 4.02) values. In contrast, NeoMTA2 and One-Fil PT showed comparatively lower variability in the recorded elemental values, with One-Fil PT exhibiting particularly low variability in oxygen content (SD = 0.55). As the SEM/EDX evaluation in the present study was intended as a descriptive material characterization approach, these findings should be interpreted cautiously and not as a comprehensive assessment of compositional heterogeneity. Representative full EDX spectra of the tested cements are shown in Figure 4 and provide qualitative profiles of the principal inorganic and radiopacifying elements detected in each material. Carbon, hydrogen, and nitrogen were not included in the present EDX assessment.

**Figure 5:**
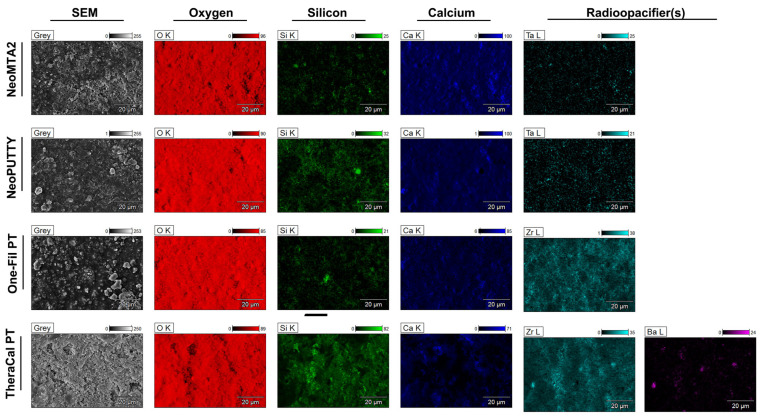
Scanning electron microscopy (SEM) and energy-dispersive X-ray spectroscopy (EDX) elemental distribution maps of calcium silicate-based cements. Representative SEM images (left) and corresponding elemental distribution maps showing oxygen (O), silicon (Si), calcium (Ca), and radiopacifiers for NeoMTA2, NeoPUTTY, One-Fil PT, and TheraCal PT. Radiopacifiers: tantalum (Ta) for NeoMTA2 and NeoPUTTY; zirconium (Zr) for One-Fil PT; zirconium (Zr) and barium (Ba) for TheraCal PT. Intensity scales (0–255 for greyscale; 0–100 for elemental maps) are shown above each image. Scale bar: 20 μm.

Elemental distribution mapping (Figure 5) supported the descriptive EDX findings and illustrated the spatial distribution of the principal detected elements on the material surfaces. Tantalum was identified in both NeoMTA2 and NeoPUTTY, whereas zirconium was detected in One-Fil PT and TheraCal PT. Barium was also detected in TheraCal PT at lower intensity. The mapping images suggested differences in the distribution patterns of these radiopacifying elements among the tested materials. Silicon was detected in TheraCal PT, and calcium was identified in all tested materials.

### 3.3. Cytotoxicity and Inflammatory Response

**Figure 6:**
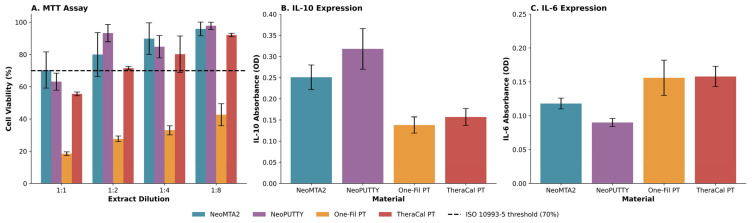
Viability and inflammatory response of human dental pulp stem cells following exposure to tested calcium silicate-based cements. (**A**) Cell viability assessed using MTT assay following 24-h exposure to material extracts at four serial dilutions (1:1, 1:2, 1:4, and 1:8). Cell viability is expressed as percentage relative to untreated control cells. Dashed line indicates 70% viability threshold according to ISO 10993-5 cytotoxicity standard. (**B**) Anti-inflammatory cytokine interleukin-10 (IL-10) secretion quantified by ELISA. (**C**) Pro-inflammatory cytokine interleukin-6 (IL-6) secretion quantified by ELISA. Cytokine expression (IL-6 and IL-10) was evaluated at 1:2 extract dilution, a concentration at which the majority of tested materials demonstrated acceptable cell viability (≥70% per ISO 10993-5) in the MTT assay. All data represent mean ± standard deviation from three independent experiments.

The MTT assay revealed concentration-dependent effects on hDPSC viability in all tested materials (Figure 6A). NeoPUTTY and NeoMTA2 showed the highest cell viability, with mean values ranging from 63.12% to 97.77% and 70.36% to 95.87% across the dilution series (1:1 to 1:8), respectively. TheraCal PT exhibited moderate viability (55.54–92.08%), while One-Fil PT showed the lowest viability across all dilutions (18.44–42.63%). The Kruskal–Wallis test revealed significant differences between materials at all dilution levels (*p <* 0.001). Mann–Whitney U tests showed that the viability of One-Fil PT was significantly lower than all other materials at every concentration tested (*p <* 0.05).

Cytokine expression analysis revealed inverse relationships between IL-6 (pro-inflammatory) and IL-10 (anti-inflammatory) markers (Figure 6B,C). NeoMTA2 and NeoPUTTY showed elevated IL-10 expression (mean: 0.251 and 0.318, respectively) alongside relatively lower IL-6 levels (mean: 0.118 and 0.090, respectively). In contrast, One-Fil PT and TheraCal PT produced higher IL-6 responses (mean: 0.156 and 0.158, respectively) and correspondingly lower IL-10 levels (mean: 0.138 and 0.157, respectively). IL-6/IL-10 ratios further clarified this pattern: tantalum-containing materials (NeoMTA2: 0.470, NeoPUTTY: 0.283) showed ratios below 0.5, while zirconium-containing materials (One-Fil PT: 1.130, TheraCal PT: 1.006) exceeded 1.0, indicating predominant pro-inflammatory responses.

## 4. Discussion

The dental materials market continues to expand with new calcium silicate-based formulations, each claiming improved performance for pulp therapy applications. Since these materials come into direct contact with the pulp, they must be biocompatible, non-toxic, have a positive effect on inflammatory mediators, and possess sufficient radiopacity to be distinguishable from surrounding tissues. In recent years, calcium-silicate-based cements have been widely used in VPT applications. Their popularity largely rests on their capacity to promote reparative dentin formation while sustaining a biological environment conducive to pulp healing [19,31]. The findings of this study revealed material-dependent differences in both physical and biological properties. TheraCal PT exhibited insufficient radiopacity and moderate cytocompatibility, while One-Fil PT showed decreased cell viability and increased IL-6 expression. These results suggest that, as previously reported, differences in chemical composition, radiopacifier type, and resin content may directly influence cellular behavior and inflammatory responses [6,17,32].

Adequate radiopacity is essential for any VPT cement, as it enables the clinician to verify material placement and monitor treatment outcomes radiographically. In this study, One-Fil PT exhibited the highest radiopacity, followed by NeoPUTTY, NeoMTA2, and TheraCal PT. This ranking is consistent with previous reports showing that zirconium- and tantalum-based radiopacifiers create more X-ray contrast compared to formulations containing barium [6,33]. The relatively low radiopacity of TheraCal PT is likely a consequence of the radiolucent resin matrix diluting the contribution of its radiopacifying agents. The findings of this study are consistent with previous studies reporting that NeoPUTTY exhibited the highest radiopacity among the similar materials evaluated, whereas NeoMTA2 showed lower radiopacity despite containing the same radiopacifier [tantalum oxide] [28]. Although the manufacturer did not provide information on the tantalum oxide content in the materials, the findings of this study indicate that NeoPUTTY contains a higher amount of tantalum than NeoMTA2 (5.71% vs. 0.32%). This difference may contribute to the radiopacity difference observed between the two materials. Furthermore, the difference may be attributed to NeoPUTTY being a premixed and ready-to-use material, while NeoMTA2 is supplied in powder-liquid form. Since powder-liquid materials can be mixed in varying ratios, differences in radiopacity may occur.

According to the ISO 6876 standard, the minimum radiopacity requirement for root canal sealing materials is ≥3 mm Al [28,29]. According to the results of this study, while all materials except TheraCal PT showed sufficient radiopacity, TheraCal PT fell well below this threshold. This result can be attributed to the material’s different radiopacifier ratio and type, as well as its light-cured nature. This is consistent with previous findings [28,29]. Based on these results, the null hypothesis regarding radiopacity was rejected. Significant differences were observed among the tested calcium silicate-based cements in terms of compliance with the ISO 6876 radiopacity requirements. It is also worth noting that calcium silicate-based cements with compositions similar to those tested in the present study are increasingly being used in flowable sealer formulations for root canal obturation, including warm compaction techniques, with favorable clinical outcomes reported in recent prospective evaluations [24]. However, the potential for apical extrusion of these materials and the resulting radiographic modifications in the periapical region should be considered as a clinical limitation, as extruded material may undergo structural and radiopacity changes over time that could complicate radiographic follow-up.

EDX analysis revealed significant differences in elemental composition and radiopacifier distribution among the tested materials. The presence of calcium, silicon, oxygen, and radiopacifying elements identified by EDX analysis was consistent with the registered formulations of the materials and previous studies on these materials [34]. EDX mapping confirmed the presence of zirconium oxide in One-Fil PT, tantalum oxide in NeoPUTTY and NeoMTA2, and barium zirconate in TheraCal PT. The type of radiopacifier directly affects both radiographic performance and hydration kinetics. In line with this, the present findings support earlier reports suggesting that zirconium and tantalum offer more stable radiographic behavior and more favorable biocompatibility than barium-based compounds [33]. High surface calcium levels are generally associated with enhanced biomineralization properties, as calcium ion release facilitates apatite nucleation and increases the odontogenic differentiation of hDPSCs [6]. According to EDX analysis, although all materials exhibited high calcium content, the highest calcium peak was observed in NeoMTA2, while the lowest was in TheraCal PT. These results are supported by previous studies indicating that MTA releases higher levels of Ca^2+^ than TheraCal PT [17,34]. A further limitation of the present study is that EDX analysis was used as a targeted compositional tool focused on the principal inorganic and radiopacifying elements relevant to the study aim, rather than as a full surface chemical characterization method. Therefore, surface reaction mechanisms and carbonate-related interpretations were beyond the scope of the present investigation.

Since VPT cements are placed in direct contact with pulp tissue, biocompatibility is a fundamental prerequisite; cytotoxic effects may compromise pulp vitality and reparative capacity [11,17]. In this study, NeoPUTTY and NeoMTA2 showed significantly higher short-term cell viability, indicating comparatively better cytocompatibility under the present experimental conditions. Consistent with our findings, previous studies have reported that NeoPUTTY exhibits the highest cell viability [11,35]. The comparatively favorable biological performance of NeoPUTTY may be associated with compositional differences among the tested materials; however, the specific contribution of individual components was not directly investigated in the present study. A recent comparative study reported that NeoPUTTY and TheraCal PT showed varying degrees of cell viability on hDPSCs depending on extract concentration, yet both maintained levels consistent with potential clinical use [6]. Previous studies have shown that TheraCal PT exhibits positive cytocompatibility at various dilutions [17,18]. Consistent with these findings, this study showed that increasing dilution ratios resulted in improved cell viability for all materials tested, reflecting a concentration-dependent cytotoxic effect. TheraCal PT demonstrated moderate short-term cytocompatibility across the tested dilutions under the present in vitro conditions.

According to the International Standard ISO 10993-5:2010, a decrease of more than 30% in cell viability is considered an indicator of potential cytotoxic effects [28]. In this study, One-Fil PT exhibited the lowest cell viability values at all concentrations (ranging from 18.44% to 42.63%) and therefore showed potential cytotoxic effects. This outcome may be associated with compositional differences among the tested materials; however, the underlying mechanisms were not directly investigated in the present study. The presence of certain radiopacifiers, resin components, or polymeric additives may potentially contribute to changes in cellular behavior, reactive oxygen species production, or inflammatory mediator release; however, further studies are needed to clarify these mechanisms. Based on these results, the second part of the null hypothesis, which suggested that there would be no significant difference in cytotoxicity among the tested putty-form calcium silicate-based cements, was rejected.

Recent studies emphasize the importance of maintaining a balance between pro- and anti-inflammatory responses in VPT cements, as this balance plays a critical role in pulp survival and regeneration [36,37]. In this study, both IL-6 and IL-10 were analyzed to assess the inflammatory response. IL-6 was selected due to its central role in regulating the acute-phase response and its strong correlation with clinical outcomes in vital pulp therapy, while IL-10 was included to evaluate the anti-inflammatory and regulatory response, allowing a more comprehensive understanding of the balance between pro- and anti-inflammatory activity [38]. The inclusion of early-phase mediators such as IL-1β is suggested for future studies. One-Fil PT and TheraCal PT exhibited relatively higher IL-6 levels, while NeoPUTTY and NeoMTA2 showed increased IL-10 expression. This indicates that the inflammatory profile of calcium silicate-based cements is material-dependent and influenced by their composition, as reflected in the distinct inflammatory patterns observed between calcium silicate-based cements and the RMCSC tested in this study.

Higher IL-6 expression may represent an early inflammatory response that supports immune cell recruitment and tissue repair; however, excessive or prolonged IL-6 levels may disrupt dentin bridge formation and tissue organization [38,39]. The increased IL-6 levels observed in One-Fil PT and TheraCal PT may reflect material-related differences in early cellular response; however, the underlying mechanisms, including possible effects of pH, ion release, or particle dissolution, were not directly investigated in the present study. Previous studies have associated increased IL-10 expression with anti-inflammatory activity [40,41]. In the present study, the higher IL-10 levels observed in NeoPUTTY and NeoMTA2 may indicate a comparatively more favorable early anti-inflammatory profile; however, no direct conclusions can be drawn regarding pulp healing, dentin bridge formation, or regenerative potential based on the present data alone. Taken together, these findings suggest that early inflammatory response patterns may be relevant when interpreting the short-term biological behavior of VPT cements alongside cell viability [42].

Although the present study demonstrated increased inflammatory response and reduced cell viability at certain concentrations, these findings should be interpreted within the limitations of in vitro models. In this context, in vivo evidence provides important complementary insights into the biological behavior of calcium silicate-based cements. A recent in vivo study demonstrated that calcium silicate-based cements, such as NeoPUTTY, may initially induce an inflammatory response on rats, which gradually subsides and is followed by tissue repair and mineralization [43]. This discrepancy may be attributed to the dynamic biological environment in vivo, where factors such as buffering capacity, vascularization, and complex cellular interactions modulate material-induced effects. Furthermore, latest in vivo investigations evaluating calcium silicate–based cements, including NeoMTA2 and NeoPUTTY, have shown that biological performance may vary depending on the implantation environment, with more pronounced alterations observed in subcutaneous tissues compared to bone. Notably, these studies emphasized that the type and distribution of radiopacifying elements may influence both tissue response and material stability, with evidence of particle release and potential systemic distribution [44]. These observations are in line with the present findings, in which differences in radiopacity and elemental composition—particularly the presence of zirconium oxide in One-Fil PT and tantalum oxide in NeoPUTTY and NeoMTA2—were associated with distinct biological and inflammatory response patterns. When considered together, these findings suggest that radiopacifier characteristics may contribute to variations in early biological responses, although the exact mechanisms underlying these effects remain to be elucidated.

The findings obtained for TheraCal PT require particularly careful interpretation. In the present study, TheraCal PT exhibited moderate short-term cell viability, yet it also induced a relatively elevated IL-6 response compared with NeoMTA2 and NeoPUTTY. This discrepancy suggests that short-term cell viability and early inflammatory response may reflect different aspects of the biological behavior of a material and do not necessarily follow the same pattern. As an RMCSC, TheraCal PT contains resin monomers including Bis-GMA and PEGDMA. Although the dual-cure polymerization mechanism of TheraCal PT has been shown to result in significantly lower residual monomer release compared with its light-cured predecessor TheraCal LC [45], leachable monomers and photoinitiators from resin-modified calcium silicate formulations may still disrupt cellular metabolism and provoke pro-inflammatory responses [34,46]. This is further supported by a recent in vivo study demonstrating that TheraCal PT induced higher inflammatory cell infiltration and inferior hard tissue formation compared with Biodentine in a rat pulpotomy model [47]. Similarly, a study comparing endodontic materials used in vital pulp therapy reported that TheraCal PT demonstrated improved in vitro cytocompatibility in hDPSCs compared with its predecessor, TheraCal LC, and exhibited biological properties comparable to Biodentine [18]. Accordingly, the coexistence of moderate short-term viability and elevated IL-6 expression observed in the present study may reflect a material that is not immediately cytotoxic at the tested dilution, yet still capable of eliciting an early inflammatory response through residual resin-related components. In contrast, One-Fil PT showed both the lowest cell viability and a pronounced pro-inflammatory response across all tested conditions, indicating a less favorable early biological profile. Although direct mechanistic evidence for this specific material remains limited, the present EDX analysis revealed a notably high zirconium oxide content (30.59 wt%) alongside minimal silicon (0.75 wt%), and the manufacturer reports a setting pH of 12.7. The combination of a high radiopacifier concentration and elevated alkalinity during setting may contribute to disruption of cellular metabolism, although the specific mechanisms underlying this response could not be determined within the scope of the present study. These findings may also be considered in a broader clinical context. Premixed calcium silicate-based cements with flowable formulations similar in composition to those tested in the present study are increasingly used in clinical applications where material contact with periapical tissues may occur. Clinical and histological evidence suggests that extruded calcium silicate-based cements can provoke short-term inflammatory reactions, and that the biological response to such contact is strongly influenced by the chemical composition of the material [48,49]. Although the present study was not designed to evaluate tissue-level reactions, the observed differences in cytokine expression and cell viability among the tested materials underscore the importance of considering the biological profile of calcium silicate-based cements alongside their physicochemical and handling properties. Favorable radiopacity or handling characteristics should not be interpreted as indicators of an equally favorable biological response, and the observed differences likely reflect broader formulation-related factors that warrant further mechanistic and long-term clinical investigation.

The present findings indicate that VPT cement selection requires an integrated assessment of radiographic properties, chemical composition, cytotoxicity, and inflammatory responses. However, the in vitro design of the present study does not fully replicate the complex biological environment of pulp tissue, and therefore the findings should be interpreted as early indicators rather than direct predictors of clinical performance. Within the limitations of this in vitro study, NeoPUTTY and NeoMTA2 exhibited comparatively more favorable early cytokine response profiles, whereas One-Fil PT and TheraCal PT induced a more pronounced early inflammatory response. One-Fil PT and NeoPUTTY demonstrated superior radiographic performance, which may be advantageous for radiographic follow-up, particularly in pediatric patients where early assessment is important. Future studies integrating longer observation periods, additional biological markers, pulp regeneration models, and controlled clinical evaluations are needed to determine the broader biological significance of these findings. In addition, the biological findings of the present study were limited to a 24-h exposure period and therefore reflect only early cellular and cytokine responses. Longer-term studies are needed to determine whether these initial findings persist over time or are associated with regenerative outcomes.

## 5. Conclusions

In conclusion, significant differences in radiographic visibility, short-term cell viability, and cytokine expression were observed among the tested calcium silicate-based cements. One-Fil PT and NeoPUTTY demonstrated superior radiographic performance, whereas NeoPUTTY and NeoMTA2 showed comparatively more favorable short-term cell viability and early cytokine response profiles under the present experimental conditions. Although all tested cements may have clinical applications, material selection should be guided by the specific physicochemical and early biological profile of each product, particularly with regard to radiopacity requirements, short-term cell viability, and cytokine response patterns. Further long-term in vivo and mechanistic studies are needed to clarify the broader biological significance of these findings.

## Figures and Tables

**Table 1:** Information on the tested dental materials.

Material	Classification, Type and Composition	Manufacturer Recommendations	Batch Number
NeoMTA2; Avalon Biomed, Houston, TX, USA	Classification: Calcium silicate cement. Type: Mineral trioxide aggregate. Composition: Powder; tricalcium silicate (<50%), dicalcium silicate (<50%), tantalum oxide (<20%), and minor amounts of calcium sulfate (<5%) and tricalcium aluminate (<5%). Liquid; water and polymers.	One scoop of powder and one or two drops of liquid, according to the desired consistency (putty or sealer) mixed 30 s to obtain homogeneous consistency. - Hand-mixed - Self-cure - Setting time: 1 h	2022010602
NeoPUTTY; Avalon Biomed, Houston, TX, USA	Classification: Calcium silicate cement. Type: Hydraulic calcium silicate cement (premixed). Composition: Tricalcium silicate (<25%), calcium aluminate (<25%), dicalcium silicate (<10%), tricalcium aluminate (<1%), calcium sulfate (<1%), tantalum oxide proprietary organic liquid and stabilizers (<50%).	- Ready-to-use paste - Self-cure - Setting time: 4 h	2021122003
One-Fil PT; Mediclus, Cheongju, Republic of Korea	Classification: Calcium silicate cement. Type: Hydraulic calcium silicate cement (premixed). Composition: Tricalcium silicate compounds (38%), zirconium oxide (42.5%), hydrophilic polymer (thickening agents).	- Ready-to-use paste - Self-cure - Setting time: 30 min	OP48T1008
TheraCal PT; Bisco, Schaumburg, IL, USA	Classification: Resin-modified calcium silicate cement (dual-cured). Type: Dual-cured resin-modified calcium silicate cement. Composition: Silicate glass-mix cement (50–75 wt%), polyethylene glycol dimethacrylate (10–30 wt%) Bis-GMA (5–10 wt%), barium zirconate (1–5 wt%), ytterbium fluoride (1–5 wt%) (CAS no. 13760-80-0), initiator (<1%).	Dispensed directly from a flowable syringe (dual syringe with automix tip). Inject the material into the mold in 1 mm increments. Light cure each increment for 20 s.	2400014951

Compositional data were obtained from manufacturers’ safety data sheets (SDS) and product documentation. Exact weight/volume percentages are not disclosed for all components; values preceded by “<“ or presented as ranges reflect the manufacturers’ reporting format. The unit of concentration (wt% or vol%) was not specified by all manufacturers.

**Table 2:** Mean radiopacity values and standard deviation (±) of the tested calcium silicate-based cements expressed as aluminum thickness equivalents (mm Al).

Material	Radiopacity (mm Al, Mean ± SD)	ISO ≥ 3 mm Al Compliance *
One-Fil PT	6.61 ± 0.32 ^a^	Yes
NeoPUTTY	6.09 ± 0.54 ^b^	Yes
TheraCal PT	1.61 ± 0.21 ^d^	No
NeoMTA2	4.34 ± 0.42 ^c^	Yes

Different superscript letters indicate statistically significant differences between groups (one-way ANOVA, Tukey post hoc test, *p <* 0.05). * Compliance with ISO 6876 (≥3 mm Al) is indicated in the third column.

**Table 3:** Elemental composition (weight%) of tested materials determined by energy-dispersive X-ray spectroscopy.

Material	O	Si	Ca	Ta	Zr	Ba
NeoMTA2	52.18 ± 1.36	3.04 ± 0.78	44.46 ± 1.76	0.32 ± 0.51	-	-
NeoPUTTY	50.79 ± 4.99	7.20 ± 2.58	36.30 ± 3.15	5.71 ± 5.72	-	-
One-Fil PT	37.37 ± 0.55	0.75 ± 0.27	31.29 ± 2.48	-	30.59 ± 1.75	-
TheraCal PT	42.80 ± 1.02	16.14 ± 4.02	12.51 ± 5.26	-	26.42 ± 1.76	2.12 ± 0.74

Values represent mean ± standard deviation. Carbon, hydrogen, and nitrogen were not quantified by EDX analysis.

**Table 4:** Elemental composition (atomic%) of tested materials determined by energy-dispersive X-ray spectroscopy.

Material	O	Si	Ca	Ta	Zr	Ba
NeoMTA2	72.78 ± 0.92	2.41 ± 0.61	24.77 ± 1.24	0.04 ± 0.06	-	-
NeoPUTTY	72.56 ± 2.77	5.97 ± 2.49	20.72 ± 0.56	0.75 ± 0.80	-	-
One-Fil PT	67.15 ± 0.95	0.77 ± 0.28	22.44 ± 1.76	-	9.64 ± 0.57	-
TheraCal PT	69.17 ± 1.28	14.87 ± 3.77	8.06 ± 3.37	-	7.49 ± 0.54	0.40 ± 0.14

Values represent mean ± standard deviation. Carbon, hydrogen, and nitrogen were not quantified by EDX analysis.

## Data Availability

The datasets generated during the current study are not publicly available due to their use in ongoing research and potential future publications but are available from the corresponding author on reasonable request.

## References

[1] Torabinejad M., Parirokh M., Dummer P.M.H. (2018). Mineral trioxide aggregate and other bioactive endodontic cements: An updated overview—Part II: Other clinical applications and complications. Int. Endod. J..

[2] Oliveira L.V., de Souza G.L., da Silva G.R., Magalhães T.E.A., Freitas G.A.N., Turrioni A.P., de Rezende Barbosa G.L., Moura C.C.G. (2021). Biological parameters, discolouration and radiopacity of calcium silicate-based materials in a simulated model of partial pulpotomy. Int. Endod. J..

[3] Camilleri J., Gandolfi M.G. (2010). Evaluation of the radiopacity of calcium silicate cements containing different radiopacifiers. Int. Endod. J..

[4] Nowicka A., Lipski M., Parafiniuk M., Sporniak-Tutak K., Lichota D., Kosierkiewicz A., Kaczmarek W., Buczkowska-Radliñska J. (2013). Response of human dental pulp capped with biodentine and mineral trioxide aggregate. J. Endod..

[5] Grech L., Mallia B., Camilleri J. (2013). Investigation of the physical properties of tricalcium silicate cement-based root-end filling materials. Dent. Mater..

[6] Bakr M.M., Shamel M., Raafat S.N., Love R.M., Al-Ankily M.M. (2024). Effect of pulp capping materials on odontogenic differentiation of human dental pulp stem cells: An in vitro study. Clin. Exp. Dent. Res..

[7] Camilleri J. (2014). Hydration characteristics of Biodentine and TheraCal used as pulp capping materials. Dent. Mater..

[8] Kang S. (2020). Mineralization-inducing potentials of calcium silicate-based pulp capping materials in human dental pulp cells. Yeungnam Univ. J. Med..

[9] Marciano M.A., Estrela C., Mondelli R.F.L., Ordinola-Zapata R., Duarte M.A.H. (2013). Analysis of the color alteration and radiopacity promoted by bismuth oxide in calcium silicate cement. Braz. Oral Res..

[10] Koutroulis A., Kuehne S.A., Cooper P.R., Camilleri J. (2019). The role of calcium ion release on biocompatibility and antimicrobial properties of hydraulic cements. Sci. Rep..

[11] Titus T., El-Sayed W., Adtani P., Zaarouor R., Nandakumar A., Elsewify T., Elemam R., Eid B. (2024). Evaluation of the antibacterial and cytotoxic effects of NeoPutty, Totalfill BC RRM Putty, and MTA Plus bioceramic root canal cements. J. Int. Dent. Med. Res..

[12] Shokouhinejad N., Razmi H., Farbod M., Alikhasi M., Camilleri J. (2019). Coronal tooth discoloration induced by regenerative endodontic treatment using different scaffolds and intracanal coronal barriers: A 6-month ex vivo study. Restor. Dent. Endod..

[13] Poggio C., Ceci M., Dagna A., Beltrami R., Colombo M., Chiesam M. (2015). In vitro cytotoxicity evaluation of different pulp capping materials: A comparative study. Arh. Hig. Rada Toksikol..

[14] Ribeiro D.A., Yujra V.Q., De Moura C.F.G., Handan B.A., de Barros Viana M., Yamauchi L.Y., Castelo P.M., Aguiar O. (2017). Genotoxicity induced by dental materials: A comprehensive review. Anticancer Res..

[15] Collado-González M., García-Bernal D., Oñate-Sánchez R., Ortolani-Seltenerich P.S., Álvarez-Muro T., Lozano A., Forner L., Llena C., Moraleda J.M., Rodríguez-Lozano F.J. (2017). Cytotoxicity and bioactivity of various pulpotomy materials on stem cells from human exfoliated primary teeth. Int. Endod. J..

[16] da Silva E.J.N.L., Zaia A.A., Peters O.A. (2017). Cytocompatibility of calcium silicate-based sealers in a three-dimensional cell culture model. Clin. Oral Investig..

[17] Rodríguez-Lozano F.J., López-García S., García-Bernal D., Sanz J.L., Lozano A., Pecci-Lloret M.P., Melo M., López-Ginés C., Forner L. (2021). Cytocompatibility and bioactive properties of the new dual-curing resin-modified calcium silicate-based material for vital pulp therapy. Clin. Oral Investig..

[18] Sanz J.L., Soler-Doria A., López-García S., García-Bernal D., Rodríguez-Lozano F.J., Lozano A., Llena C., Forner L., Guerrero-Gironés J., Melo M. (2021). Comparative biological properties and mineralization potential of 3 endodontic materials for vital pulp therapy. J. Endod..

[19] Tsuchiya K., Sauro S., Sano H., Matinlinna J.P., Yamauti M., Hoshika S., Toida Y., Islam R., Tomokiyo A. (2025). Clinical applications and classification of calcium silicate-based cements based on their history and evolution: A narrative review. Clin. Oral Investig..

[20] Antunes T.B.M., Janini A.C.P., Pelepenko L.E., Abuna G.F., Paiva E.M., Sinhoreti M.A.C., Raimundo I.M., Gomes B.P.F.A., de-Jesus-Soares A., Marciano M.A. (2021). Heating stability, physical and chemical analysis of calcium silicate-based endodontic sealers. Int. Endod. J..

[21] Al-Haddad A., Che Ab Aziz Z.A. (2016). Bioceramic-based root canal sealers: A review. Int. J. Biomater..

[22] Küçükkaya Eren S. (2023). Clinical applications of calcium silicate-based materials: A narrative review. Aust. Dent. J..

[23] Wang X., Xiao Y., Song W., Ye L., Yang C., Xing Y., Yuan Z. (2023). Clinical application of calcium silicate-based bioceramics in endodontics. J. Transl. Med..

[24] Zamparini F., Spinelli A., Cardinali F., Ausiello P., Gandolfi M.G., Prati C. (2023). The use of premixed calcium silicate bioceramic sealer with warm carrier-based technique: A 2-year study for patients treated in a master program. J. Funct. Biomater..

[25] Camilleri J. (2020). Classification of hydraulic cements used in dentistry. Front. Dent. Med..

[26] (2025). Dentistry — Endodontic Sealing Materials.

[27] (2009). Biological Evaluation of Medical Devices — Part 5: Tests for in Vitro Cytotoxicity.

[28] Altinok-Uygun L., Sözen E. (2024). Evaluation of the radiopacity of pulp capping materials. Necmettin Erbakan Univ. Dent. J..

[29] Corral C., Negrete P., Estay J., Osorio S., Covarrubias C., de Oliveira Junior O.B., Barud H. (2018). Radiopacity and chemical assessment of new commercial calcium silicate-based cements. Int. J. Odontostomatol..

[30] Pedano M.S., Li X., Yoshihara K., Van Landuyt K., Van Meerbeek B. (2020). Cytotoxicity and bioactivity of dental pulp-capping agents towards human tooth-pulp cells: A systematic review of in vitro studies and meta-analysis of randomized and controlled clinical trials. Materials.

[31] Camilleri J., Sorrentino F., Damidot D. (2013). Investigation of the hydration and bioactivity of radiopacified tricalcium silicate cement, Biodentine and MTA Angelus. Dent. Mater..

[32] Novotná B., Holík P., Morozova Y., Rosa M., Galandáková A., Langová K. (2024). Evaluation of cytotoxicity of the dental materials TheraCal LC, TheraCal PT, ApaCal ART and Biodentine used in vital pulp therapy: In vitro study. Dent. J..

[33] Zhou J., Ou M.H., Wei X.L., Lan X.L., Lan B.Y., Chen W.J., Song S.J., Chen W.X. (2022). The role of different macrophages-derived conditioned media in dental pulp tissue regeneration. Tissue Cell.

[34] Küden C., Karakaş S.N., Batmaz S.G. (2022). Comparative chemical properties, bioactivity, and cytotoxicity of resin-modified calcium silicate-based pulp capping materials on human dental pulp stem cells. Clin. Oral Investig..

[35] Hakeem J., Shamaa A., Mohammed S.S. (2023). Effect of premixed bioceramic putty and mineral trioxide aggregate on human fibroblasts (an in vitro study). Egypt. Dent. J..

[36] Goldberg M. (2008). In vitro and in vivo studies on the toxicity of dental resin components: A review. Clin. Oral Investig..

[37] Giraud T., Jeanneau C., Rombouts C., Bakhtiar H., Laurent P., About I. (2019). Pulp capping materials modulate the balance between inflammation and regeneration. Dent. Mater..

[38] Qiao L., Zheng X., Xie C., Wang Y., Ye L., Zhao J., Liu J. (2025). Bioactive materials in vital pulp therapy: Promoting dental pulp repair through inflammation modulation. Biomolecules.

[39] Chung M., Lee S., Chen D., Kim U., Kim Y., Kim S., Kim E. (2019). Effects of different calcium silicate cements on the inflammatory response and odontogenic differentiation of lipopolysaccharide-stimulated human dental pulp stem cells. Materials.

[40] Jiang S., Lin Y., Huang D., Tan X. (2025). The effect of the inflammatory microenvironment on odontogenic differentiation of dental pulp stem cells. Stem Cells Dev..

[41] Farges J.C., Carrouel F., Keller J.F., Baudouin C., Msika P., Bleicher F., Staquet M.-J. (2011). Cytokine production by human odontoblast-like cells upon Toll-like receptor-2 engagement. Immunobiology.

[42] Mutar M.T., Mahdee A.F. (2024). Different pulp capping agents and their effect on pulp inflammatory response: A narrative review. Saudi Dent. J..

[43] Silva E.C.A., Pradelli J.A., da Silva G.F., Cerri P.S., Tanomaru-Filho M., Guerreiro-Tanomaru J.M. (2024). Biocompatibility and bioactive potential of NeoPUTTY calcium silicate-based cement: An in vivo study in rats. Int. Endod. J..

[44] Janini A.C.P., Pelepenko L.E., Moraes B.F., dos Santos V.A.B., Barros-Costa M., dos Santos I.A.M., de Souza Batista F.R., de Aguiar Silveira Meira J., Matsumoto M.A., da Silva N.A. (2025). Chemical and in vivo analyses of calcium silicate-based materials in bone and connective tissues. Int. Endod. J..

[45] Park S.H., Ye J.R., Asiri N.M., Chae Y.K., Choi S.C., Nam O.H. (2024). Biocompatibility and bioactivity of a dual-cured resin-based calcium silicate cement: In vitro and in vivo evaluation. J. Endod..

[46] Hernández-Cabanillas J.C., Hardan L., Cuevas-Suárez C.E., Olivares-Acosta I., Dang A.T., Tosco V., Kharouf N., Lukomska-Szymanska M., Haikel Y., Bourgi R. (2025). Comparative evaluation of the physicochemical and biological properties of calcium silicate-based pulp capping materials. Front. Dent. Med..

[47] Gok T., Cankaya G., Eroksuz Y., Akdeniz Incili C., Karadeniz Saygili S. (2025). Effect of TheraCal PT and Biodentine on inflammatory cell infiltration and hard tissue formation after pulpotomy in inflamed or healthy rat molars. Clin. Oral Investig..

[48] Zamparini F., Lenzi J., Duncan H.F., Spinelli A., Gandolfi M.G., Prati C. (2024). The efficacy of premixed bioceramic sealers versus standard sealers on root canal treatment outcome, extrusion rate and post-obturation pain: A systematic review and meta-analysis. Int. Endod. J..

[49] Takahara S., Edanami N., Ibn Belal R.S., Yoshiba K., Takenaka S., Ohkura N., Yoshiba N., Gomez-Kasimoto S., Noiri Y. (2025). An evaluation of the biocompatibility and chemical properties of two bioceramic root canal sealers in a sealer extrusion model of rat molars. J. Funct. Biomater..

